# Phenex: Ontological Annotation of Phenotypic Diversity

**DOI:** 10.1371/journal.pone.0010500

**Published:** 2010-05-05

**Authors:** James P. Balhoff, Wasila M. Dahdul, Cartik R. Kothari, Hilmar Lapp, John G. Lundberg, Paula Mabee, Peter E. Midford, Monte Westerfield, Todd J. Vision

**Affiliations:** 1 National Evolutionary Synthesis Center, Durham, North Carolina, United States of America; 2 Department of Biology, University of North Carolina at Chapel Hill, Chapel Hill, North Carolina, United States of America; 3 Department of Biology, University of South Dakota, Vermillion, South Dakota, United States of America; 4 Academy of Natural Sciences, Philadelphia, Pennsylvania, United States of America; 5 Institute of Neuroscience, University of Oregon, Eugene, Oregon, United States of America; BC Centre for Excellence in HIV/AIDS, Canada

## Abstract

**Background:**

Phenotypic differences among species have long been systematically itemized and described by biologists in the process of investigating phylogenetic relationships and trait evolution. Traditionally, these descriptions have been expressed in natural language within the context of individual journal publications or monographs. As such, this rich store of phenotype data has been largely unavailable for statistical and computational comparisons across studies or integration with other biological knowledge.

**Methodology/Principal Findings:**

Here we describe Phenex, a platform-independent desktop application designed to facilitate efficient and consistent annotation of phenotypic similarities and differences using Entity-Quality syntax, drawing on terms from community ontologies for anatomical entities, phenotypic qualities, and taxonomic names. Phenex can be configured to load only those ontologies pertinent to a taxonomic group of interest. The graphical user interface was optimized for evolutionary biologists accustomed to working with lists of taxa, characters, character states, and character-by-taxon matrices.

**Conclusions/Significance:**

Annotation of phenotypic data using ontologies and globally unique taxonomic identifiers will allow biologists to integrate phenotypic data from different organisms and studies, leveraging decades of work in systematics and comparative morphology.

## Introduction

The manifestation of evolution at the organismal level is the phenotype, the set of observable traits inhering in an individual organism as a result of the interaction of heredity, environmental influences, and developmental processes. Biologists from different subdisciplines have approached the study of phenotypes in different ways. The unfolding of the phenotype from a fertilized egg is at the core of developmental biology, the inference of gene function through the phenotypic effect of allelic differences is a major focus of genetics, and using phenotypes to inform and interpret phylogenies in living and fossil organisms is at the core of systematics and comparative biology.

Despite the centrality of the phenotype to so much of biology, traditions for communicating information about phenotypes are idiosyncratic to different disciplines. Phenotypes seem to elude standardized descriptions due to the variety of traits that compose them and the difficulty of capturing the complex forms and subtle differences among organisms that we can readily observe. Consequently, phenotypes are refractory to attempts at data integration that would allow computational analyses across studies and study systems [1]. Here, we address this problem at its root by development of a configurable software tool that employs standard ontologies and syntax to create computable phenotype annotations.

Ontologies have become a foundational technology for establishing shared semantics, and, more generally, for capturing and computing with biological knowledge. An ontology is a type of structured vocabulary in which the terms and the logical relationships that hold between them are well-defined. Ontologies are particularly well-suited to providing machine-readable context for polymorphous and qualitative biological data, as exemplified by the success of the Gene Ontology (GO) [2]. By describing gene products from many different organisms with GO terms, a very broad scientific community has been able to communicate knowledge about gene function in a way that is simultaneously readable by humans and by machines, and innumerable applications have been developed to exploit these properties [3]. Ontologies have been and are being developed for many different domains of knowledge in the life science under the umbrella of the Open Biological and Biomedical Ontologies (OBO) Consortium [4]. The OBO Consortium promotes a shared set of best practices, including the accessibility of the ontologies through common formats and repositories.

The genetic model organism community, in particular, has pioneered the application of ontologies to phenotype data, specifically for describing the extraordinary diversity of shape, size, position, composition, etc. in the observable physical characteristics in mutant genotypes relative to the wild type [4]. The syntactic convention adopted by the OBO Consortium for describing such phenotypes is called the ‘Entity-Quality’ (EQ) formalism [5], [6]. EQ associates an entity term drawn from an organism-specific ontology (e.g. *fin*, *vertebra*, or *skull* from the Zebrafish Anatomy Ontology) with a quality term from the generic Phenotype and Trait Ontology (PATO). PATO terms describe the quality or value of some attribute of the entity (such as its *color*, *size*, *shape*, or *count*). EQ syntax has a number of desirable computational features [7]: it yields compact representations because one need only specify qualities for which a given phenotype description has a value; there are no arbitrary limits on the number of qualities that can be associated to an entity; it supports complex queries because terms come from hierarchically structured ontologies; and separating entity (E) and quality (Q) terms permits development of a small generic quality ontology orthogonal to any number of domain-specific entity ontologies. Specialized software has been developed to assist human curators in annotating the phenotypes of mutant genotypes using EQ syntax, such as the software tool Phenote, which is in use at the ZFIN, WormBase, and Flybase databases [4].

Evolutionary biologists have compared and described phenotypic differences among species—extinct and extant—in the systematics and paleontological literature for many decades [8]. One of the most common and also most formalized approaches within evolutionary biology is in the field of phylogenetic systematics, where the variable organismal features (characters) and their variants in different taxa (character states) are itemized and given numeric codes in a character-by-taxon matrix. Importantly, the character and character state descriptions themselves are expressed in natural language. In the character-by-taxon matrix, the rows index taxa, or operational taxonomic units (OTUs), and the columns index characters. Individual cells contain a numeric code for a particular character state. For example, a group of species may vary in the character ‘opercle shape’, with some species exhibiting the character state ‘triangular’ and represented by a ‘0’ in the matrix, whereas other species may exhibit another character state ‘round’ and be represented by a ‘1’ in the matrix. Character-by-taxon matrices are analyzed for the purpose of recovering phylogenetic relationships or for examining patterns of character change on a given phylogeny.

Character-by-taxon matrices can be created or edited in software programs such as Mesquite [9] and MacClade [10] or web-based systems such as Morphobank (http://www.morphobank.org/) and MX (http://purl.org/NET/mx-database). The most common representation of these data is in NEXUS format [11]. Neither NEXUS, nor several other standards that have been developed for descriptive species data [12], [13], has built-in support for linking ontology terms to the text strings used to describe characters, character states, and taxonomic names. Ramírez *et al*. [14] proposed a system for linking specimen images to phylogenetic data sets via an anatomical ontology, but it is not yet general practice and is not supported by the commonly used software tools and standards. While adequate for human interpretation, free text strings are seldom unambiguous and are semantically opaque to computers, precluding computational comparison of phenotype data across multiple evolutionary studies and the integration of these data with other biological knowledge on the web.

Here we describe a new software tool for phenotypic data curation. The software, called Phenex, was developed in the context of the Phenoscape project (http://phenoscape.org) in support of using the EQ formalism to transform a large body of morphologic character descriptions from the legacy systematics literature into computable phenotype annotations [8], [15], [16]. Phenoscape focuses on the ichthyological systematics literature to link evolutionary variation in fish morphology to the rich store of phenotype data from genetic studies in zebrafish [6]. The aim of establishing such linkages is to facilitate work on fundamental questions at the intersection of evolutionary and developmental biology, including the discovery of candidate genes underlying observed evolutionary changes, analyses of genetically correlated traits, and discovering evolutionary transitions that mirror the phenotypic effects of gene mutations. A companion paper describes the standards that have been developed within the Phenoscape project for the curation process [17].

## Methods

### Software Design

The initial requirements for Phenex grew out of early experiences using Phenote, the mutant phenotype curation tool in use by several model organism databases [4]. Phenex was created using the same application framework as Phenote, but the user interface in Phenex differs in being explicitly oriented around the ability to easily manage large character-by-taxon matrices. Data entry is compartmentalized between separate panels for characters and states, phenotypes, matrix, and specimens list.

Phenex was developed using agile development methodology—regular iterations of software improvement based upon continuous feedback from users. The requirements refinement process was driven on one hand through continuous input from individual curators in the course of their work, and on the other hand from training morphology experts as new users during periodic data curation jamborees. As the domain experts had varying levels of familiarity with ontologies, the jamborees allowed us to observe how intuitive the tool's user interface is in supporting the overall curation workflow and the application of EQ syntax to character data.

### Implementation

Phenex is a desktop application that will run on systems with Java 5 or higher. It makes heavy reuse of the application framework developed for the OBO-Edit ontology editor [18], which provides the ontology object model, ontology reading capabilities, and interface framework. Like OBO-Edit, Phenex is open source, released under the MIT license (http://www.opensource.org/licenses/mit-license.php). The Phenex homepage (http://phenex.sourceforge.net/) includes links to download the latest release as well as user documentation. Source code is available from the Phenex project Sourceforge repository (http://sourceforge.net/projects/phenex/). This paper describes version 1.0.

### Input and Output

**Figure 1:**
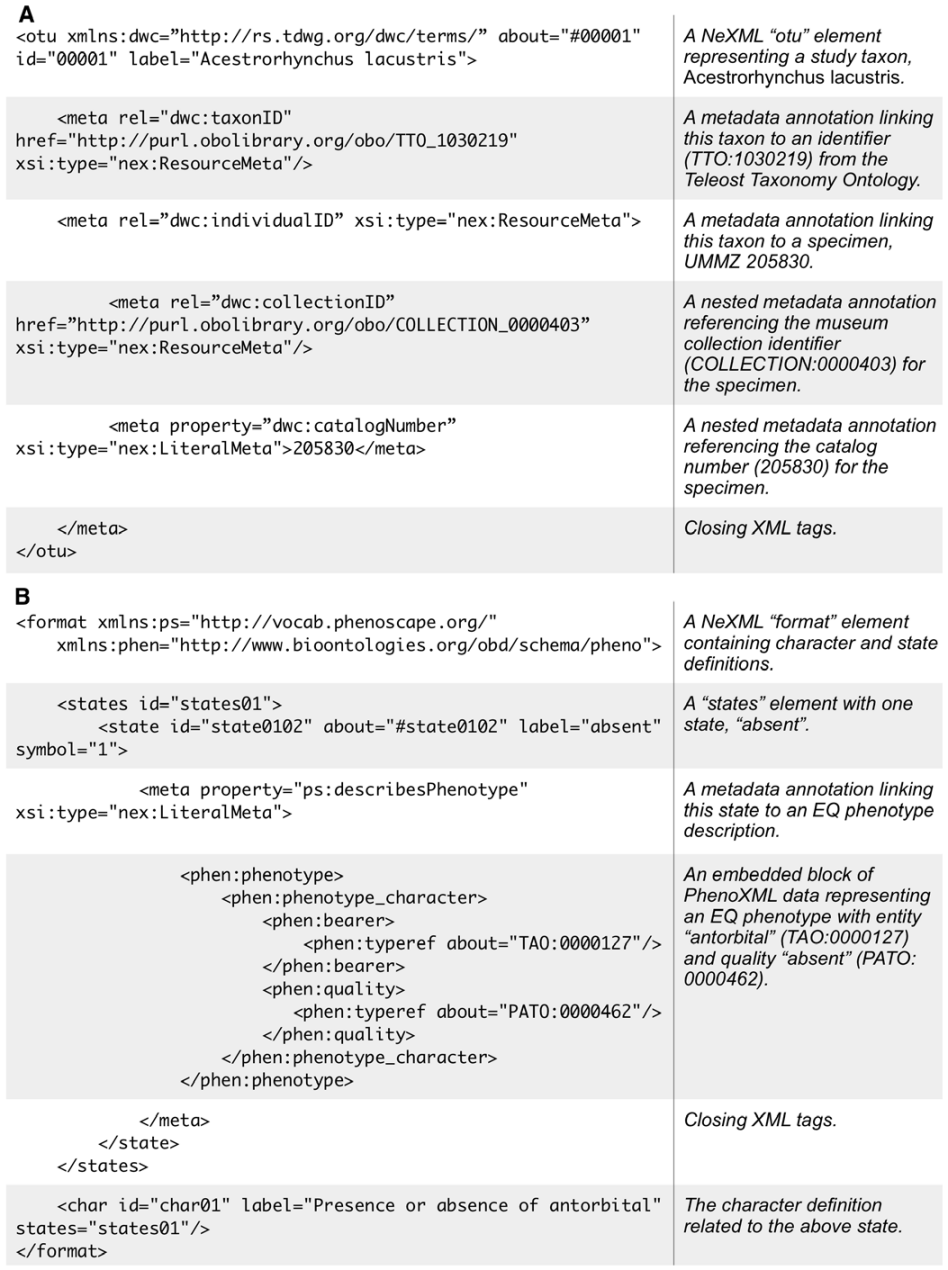
NeXML fragments demonstrating embedded Phenex annotations. A. A taxon. B. A character and character state.

For interoperability with legacy tools and data, Phenex can import lists of taxa, characters and character states, as well as character-by-taxon matrices, from NEXUS, the most widely-used file format in systematics [11]. However, the native Phenex file format is NeXML (http://www.nexml.org), an XML-based phylogenetic data exchange standard inspired by NEXUS. NeXML provides a built-in means to embed ontology-based annotations such as EQ statements within standard character-by-taxon matrix data. These embedded annotations adhere to the RDFa syntax for the attachment of metadata to data elements within XML documents (http://www.w3.org/TR/rdfa-syntax/). Where possible, metadata relations, which designate the type of relationship between data and metadata object, are drawn from from existing standards to facilitate repurposing of the annotations for other applications (Table 1). Terms from the Darwin Core metadata standard (http://rs.tdwg.org/dwc/) are used to link taxa to unique identifiers for taxa and specimens (Figure 1A), and terms from the Dublin Core Metadata Initiative (http://dublincore.org) are used for general document information such as identification of the data curator. EQ phenotypes are attached to character states and are serialized using the PhenoXML schema (http://www.fruitfly.org/~cjm/obd/formats.html), a standard developed by the OBO community for representing Entity-Quality phenotype descriptions (Figure 1B).

**Table 1:** Metadata identifiers and XML elements used by Phenex to embed annotations with NeXML documents.

Identifier	Namespace	Source	Usage
creator	http://purl.org/dc/terms/	Dublin Core	Relates NeXML document to curators of content.
references	http://purl.org/dc/terms/	Dublin Core	Relates NeXML document to publication source.
description	http://purl.org/dc/terms/	Dublin Core	Relates NeXML document to contents of Phenex “Publication Notes” field.
taxonID	http://rs.tdwg.org/dwc/terms/	Darwin Core	Relates each NeXML taxon to the OBO identifier used for the Phenex “valid name”.
individualID	http://rs.tdwg.org/dwc/terms/	Darwin Core	Relates each NeXML taxon to each specimen entry.
collectionID	http://rs.tdwg.org/dwc/terms/	Darwin Core	Relates each specimen entry to a museum collection OBO identifier.
catalogNumber	http://rs.tdwg.org/dwc/terms/	Darwin Core	Relates each specimen entry to an accession code for a museum collection.
comment	http://www.w3.org/2000/01/rdf-schema#	RDF-Schema	Relates NeXML taxon, character, and state elements to curator comments.
hasMatrixName	http://vocab.phenoscape.org/	Phenoscape	Relates each NeXML taxon to the Phenex “matrix name”.
inFigure	http://vocab.phenoscape.org/	Phenoscape	Relates NeXML taxon, character, and state elements to figure references.
describesPhenotype	http://vocab.phenoscape.org/	Phenoscape	Relates NeXML state elements to a block of PhenoXML data representing EQ phenotypes.
phenotype	http://www.bioontologies.org/obd/schema/pheno	PhenoXML	Represents a collection of EQ statements.
phenotype_character	http://www.bioontologies.org/obd/schema/pheno	PhenoXML	Represents a single EQ statement.
bearer	http://www.bioontologies.org/obd/schema/pheno	PhenoXML	Represents the entity component of an EQ statement.
quality	http://www.bioontologies.org/obd/schema/pheno	PhenoXML	Represents the quality component of an EQ statement.
related_entity	http://www.bioontologies.org/obd/schema/pheno	PhenoXML	Represents the related entity component of a relational EQ statement.
typeref	http://www.bioontologies.org/obd/schema/pheno	PhenoXML	Represents a reference to a particular OBO ontology term or post-composition.

Identifiers in the http://vocab.phenoscape.org/ namespace are intended to be replaced with community standards as they become available.

## Results

### Systematic characters and the EQ formalism

**Figure 2:**
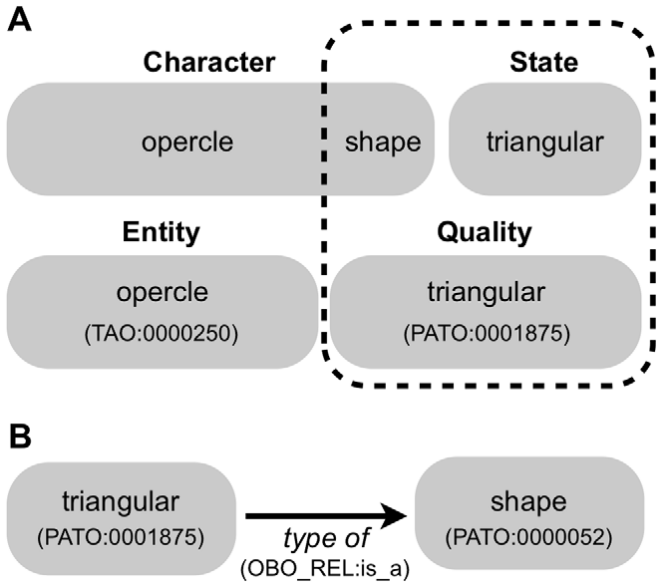
Correspondence between Entity-Quality statements and evolutionary characters. A. Comparison of the structure of phenotypic descriptions using character-character state vs. Entity-Quality ( =  ‘Phenotype’) syntaxes. B. The defined relationship between an attribute quality type (*shape*) and a value quality type (*triangular*) within the Phenotype and Trait Ontology (PATO).

The core function of Phenex is to allow users to construct EQ statements, i.e. ‘phenotypes’, corresponding to each unique character state present in a character-by-taxon matrix. While character/character state definitions and EQ statements are both used to represent phenotype descriptions, there is not a one-to-one mapping between the two. A character (such as ‘opercle shape’), typically includes both an entity term (*opercle*) as well as the particular variable attribute of that entity (*shape*) (Figure 2A, top). The character state (in this case, ‘triangular’), describes the value that the attribute takes in some specimen or taxon. In contrast, the attribute is implicit in the equivalent EQ statement (Figure 2A, bottom), because in the PATO subtype hierarchy value qualities are specific kinds of attribute qualities [5], [6]. For example, something that can be *triangular* in PATO is by necessity a type of *shape* (Figure 2B). Qualifying which attribute of the entity is variable in an EQ statement would hence be redundant.

The EQ representation of a description such as “the opercle is approximately triangular in shape” [19] is formally represented as a type of the quality *triangular* which *inheres_in* the entity *opercle*, More precisely, the EQ phenotype *is_a* type of quality term *triangular* (PATO:0001875) from PATO, that *inheres_in* the entity term *opercle* (TAO:0000250) from the Teleost Anatomy Ontology (TAO). The *is_a* and *inheres_in* relations are derived from the OBO Relations Ontology ([20]; http://obofoundry.org/cgi-bin/detail.cgi?id=relationship) and from proposed extensions to this ontology (http://www.bioontology.org/wiki/index.php/RO:Main_Page).

In applying the EQ formalism to systematic characters, it is helpful to distinguish several categories of characters that are commonly found in the systematics literature [17]. A character may fall into more than one of these categories. In describing these below, we use the following abbreviations: E, entity; Q, quality; C, count; RE, related entity. We describe the support that was built into Phenex for the representation of these characters in subsequent sections.

*Monadic (non-relational) characters and states* are those that involve single entities or anatomical structures. These characters are annotated with quality terms from PATO that are children of *quality of single physical entity* (PATO:0001237), such as *shape*, *size*, and *structure* and their children. For example, the caudal fin is described as having a deeply forked margin in some gonorynchiform fishes [21]. This is annotated as: E: *caudal fin*; Q: *bifurcated*.

*Relational characters and states* are those that involve two entities or anatomical structures. Such characters are annotated with quality terms from PATO that are children of *quality of related physical entities* (PATO:0001238) and these quality terms describe a phenotype that exists between two entities. For example, the two bones of the caudal fin (hypural 2 and hypural 3) are described as fused in some characiform taxa [22]. This is annotated as: E: *hypural 2*, Q: *fused_with*, RE: *hypural 3*.

*Composite character states* involve multiple phenotypes for a single character state. These character states can be monadic or relational. Systematists often describe multiple features in a character state to capture anatomical complexity, to document non-independent properties of a single anatomical entity, or to represent what is assumed to be a ‘character complex’. For example, the shape of the caudal fin of catfishes is described with the following three states [23]: forked with pointed lobes (0); forked with rounded lobes (1); scarcely emarginate to rounded (2). Each of these states, however, requires three distinct EQ phenotypes, e.g. state 0 is annotated with: E: *caudal fin*, Q: *bifurcated*, E: *caudal fin upper lobe*, Q: *sharp* and E: *caudal fin lower lobe*, Q: *sharp*.

*Quantitative characters* provide a literal value for a variable phenotypic feature (e.g., size, area, count). For example, characters involving counts of entities are annotated using the *count* quality and the literal values are recorded in the count field. Variation in meristics such as vertebral number are commonly described across species, e.g. [24]: state 0: 40–42; state 1: 43; state 2: 44–45. State 0 is annotated as: E: *vertebra*, Q: *count*, C: 40–42.

### Use of the Phenex software for EQ annotation of phenotypes

#### Loading ontologies

Phenex can load any OBO-formatted ontology available on the web or in a local file, and it uses these ontologies for nearly every data type. Using the ontology configuration panel, users can specify URLs from which Phenex should load ontology terms. The most recent version of each ontology is loaded each time Phenex is launched. Users can specify a term filter for each type of entry field, which determines the collection of terms provided as autocomplete suggestions for that field. These filters are commonly used to specify that an entry field uses terms which are drawn from a particular namespace or ontology subset (known as a “slim” in OBO parlance), or have specific relationships to other terms, so that only relevant suggestions are provided to the user. For example, in the Phenoscape configuration of Phenex, the entity field only draws on terms from the Teleost Anatomy Ontology, Spatial Ontology, or Gene Ontology Biological Process namespace.

#### Interface

**Figure 3:**
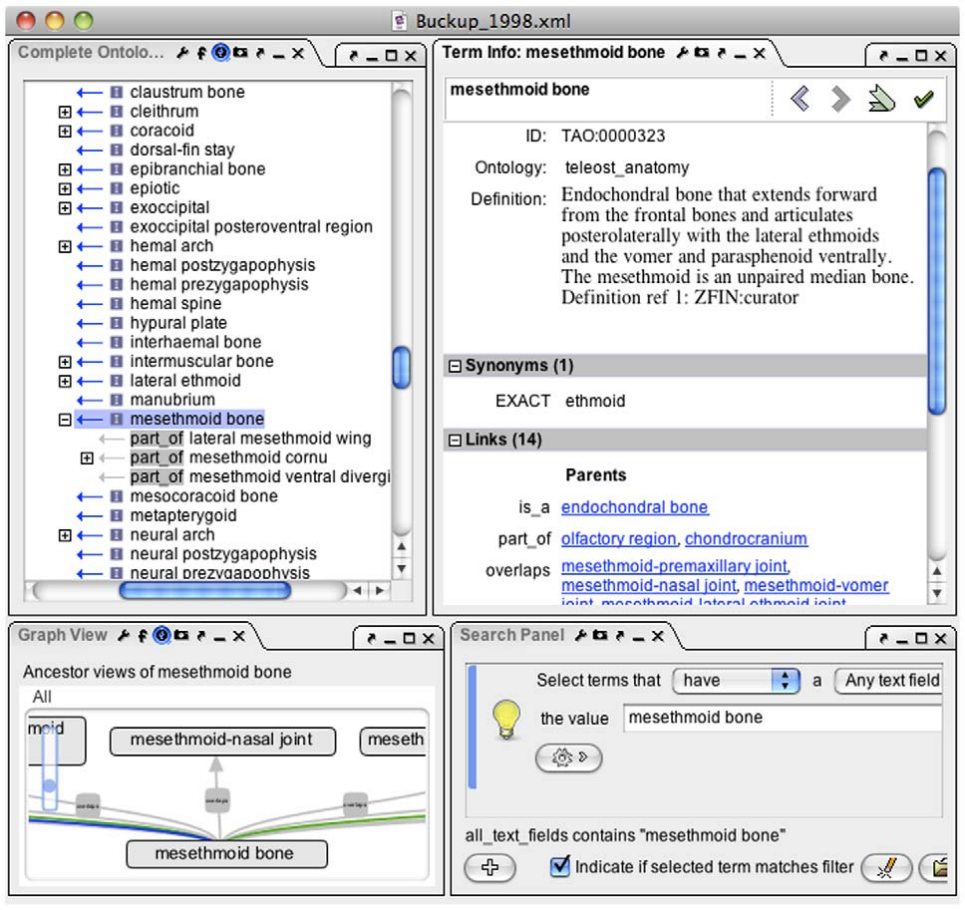
Phenex screenshot of window configured with panels for browsing and searching of ontology terms and relationships. Note that users can configure the position and size of each panel on the fly. See text for interface details of each panel; the window shows data from a publication [31] curated by the Phenoscape project.

**Figure 4:**
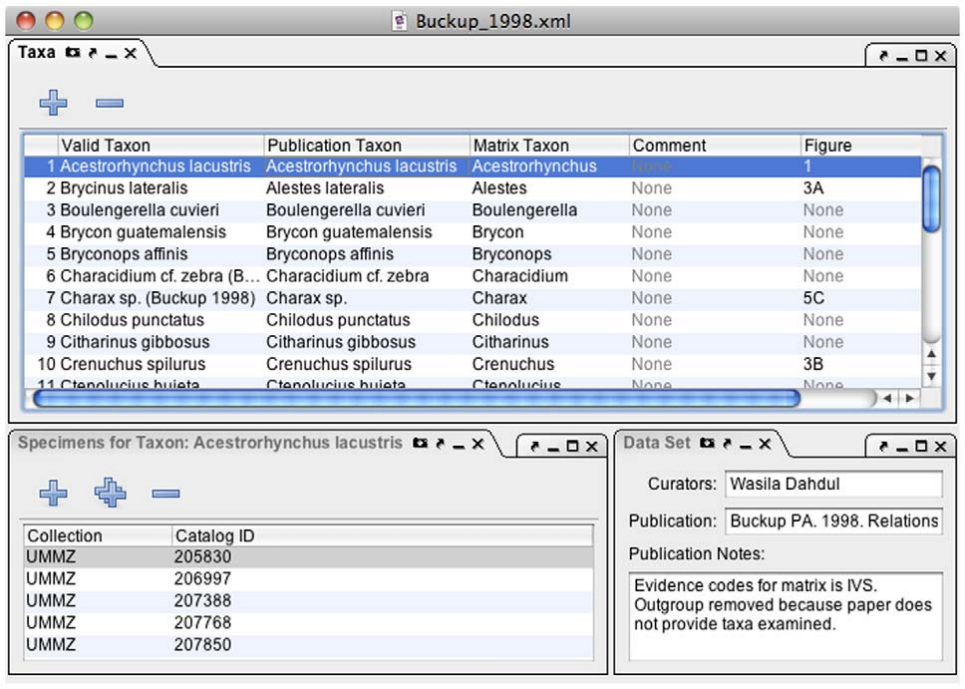
Phenex screenshot of window configured with panels for editing of taxon lists, voucher specimens, and publication information.

**Figure 5:**
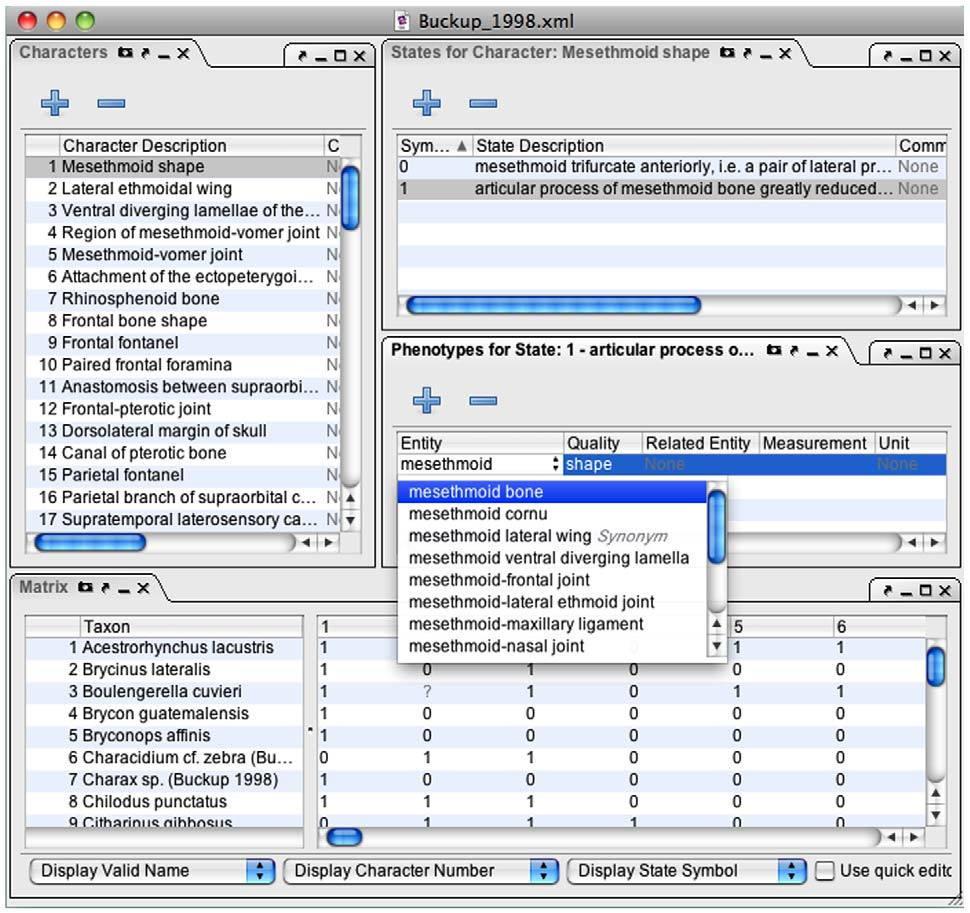
Phenex screenshot of window configured with panels for editing of character and character states data, phenotypes (i.e. EQ statements), and character-by-taxon matrix.

Phenex provides a straightforward and familiar graphical user interface (GUI) to evolutionary biologists who work routinely with lists of taxa, characters, character states, and character-by-taxon matrices. In this way, it differs from the closely related Phenote software, which is commonly used for EQ annotation of mutant phenotypes in the genetics community [4]. Both Phenote and Phenex inherit a flexible, modular interface design from the OBO-Edit application framework. The primary GUI components in Phenex include newly developed panels for editing taxa, specimens, characters, character states, character-by-taxon matrices, EQ statements, and literature citations, as shown in Figure 3, Figure 4 and Figure 5 and described below. In addition, Phenex reuses several GUI components provided by OBO-Edit and Phenote that assist users to quickly find and evaluate ontology terms. These include graphical displays of term relationships (‘Complete Ontology Tree View’), a sophisticated term ‘Search Panel’, and a textual ‘Term Info’ panel that displays synonyms, definitions, and relationships (Figure 3). All panels displaying ontology term information are updated with the currently selected term in the primary editing interface.

#### Taxa Panel

Phenex allows the curator to relate any obsolete or otherwise invalid taxonomic names used by the original authors to currently valid names, as represented by a taxonomic ontology (Midford et al. in prep.). The taxon name used in the original publication and the currently valid name correspond to ‘Publication Taxon’ and ‘Valid Taxon’, respectively, in Figure 4. Also, since many publications report phenotypes for higher taxa (genera, families, etc.) rather than species, Phenex allows entry of a ‘Matrix Taxon’ that corresponds to the set of species with specimens that were actually examined. Comments and illustrative figure references may be associated with a particular taxon entry.

#### Specimens Panel

Phenex allows the user to record information about the specimens reported in the original publication. It is standard and often required practice for publications in systematics to include a list of the voucher specimens on which phenotypic observations were based. Voucher specimens are deposited and catalogued (or registered) in the permanent collections of natural history museums. Future investigators may reexamine these physical specimens to validate or extend the original observations, or to retrieve additional information that pertains to each voucher, such as the collection locality. Thus, the information about specimens is part of the evidence for phenotype annotations, and as such important for the reusability of the data. Phenex facilitates selection of a museum or institution code from a look-up table and manual entry of the catalog number for each voucher specimen reported in the publication (Figure 4). As museums increasingly have publicly accessible digital collection databases, the combination of museum code and catalog number allows users to look up additional metadata for such specimens.

#### Characters and States-for-Character Panels

The ‘Characters’ panel allows the user to manually enter a free-text description for each character in an auto-incremented numbered list. This reflects the typical practice in systematics publications to report the characters in a numbered list, where the numbers index the columns of the character-by-taxon matrix. Besides entering the free text, a Comment field can be used, for example, to provide English translation of the character text when the original publication is in another language (Figure 5), and the Figure field can hold references to any illustrative figures for the specific character. The ‘States for Character’ panel (Figure 5) provides for manual entry of the symbols used for the states of a specific character. Typically the states are ‘0’ or ‘1’, but sometimes authors use ‘a’, ‘b’ or other variations. Similar to the ‘Characters’ panel, the user can enter free-text descriptions of the character state, comments, and figure references.

#### Phenotypes Panel

The ‘Phenotypes’ panel enables curators to create Entity-Quality statements for each character state (Figure 5). Ontology terms are selected for the Entity, Quality, and (optionally) Related Entity fields, thus supporting annotation of both monadic and relational characters. An ‘Add Phenotype’ button (+ on Phenotypes panel, Figure 5) facilitates the entry of multiple phenotypes for a single character state, thus supporting annotation of composite characters. The values for quantitative characters may be entered using the fields for counts and measurements. Units (e.g. millimeters, milligrams, etc.) are recorded within the ontology-enabled unit field.

Phenex fully supports post-composition of entity terms that are not in the chosen anatomy (or any other entity) ontology. Post-composed terms are new terms created from the semantic intersections of existing terms in one or more ontologies [25]. For example, many skeletal structures vary in the presence, shape, and size of their “margins”, “processes”, or “regions”. For example, variation in a skull bone, the epiotic, is described as [26]: ‘Epiotic process, pointed (0) or bifurcated distally (1)’. ‘Epiotic process’ is represented as the post-composition *process* that is *part_of epiotic*. Post-composition allows one to use such phenotypes in EQ statements without exhaustively enumerating all such terms in the ontology through ‘pre-composition’. Phenex also allows a curator to add human-readable comments to a post-composition, for example to express difficulties in interpretation of the published character description.

### Application of Phenex to Evolutionary Phenotypes

One of the fundamental steps in linking evolutionarily variable phenotypes to phenotypes of genetic mutants is to express both kinds of phenotypes using the same formalism. Demonstrating the power of this integrative approach is the aim of the Phenoscape project, which uses the EQ syntax as the common formalism, OBO ontologies as the source of mutually understood terms and relations, and the Ostariophysi as the taxonomic focus. The Ostariophysi are a large clade of teleost fishes with a rich literature on comparative morphology. The clade also includes zebrafish (*Danio rerio*), a model organism for developmental genetics with an abundance of EQ phenotype data already available from the Zebrafish Information Network (ZFIN) database.

The Phenoscape curation workflow, of which Phenex is a critical piece, resulted in 12,861 EQ annotations or ‘phenotypes’ for a large collection (47) of evolutionary character matrices [17]. For Phenoscape, Phenex was configured to load the Teleost Anatomy Ontology (TAO), Phenotype and Trait Ontology (PATO), and Teleost Taxonomy Ontology (TTO, Midford et al., in prep.) as well as the Gene Ontology (GO), the Spatial Ontology (BSPO), the Relations Ontology (RO), the Evidence Code Ontology (ECO), the Unit Ontology (UO), and a list of museum identifiers. All the ontologies are available from the repository of the OBO Library, while the list of museum identifiers is available from Phenoscape (http://phenoscape.org/vocab/fish_collection_abbreviation.obo). Phenex is easily configured to generate EQ statements for a different set of organisms by simply loading different anatomy and taxonomy ontologies upon startup.

One the strengths of Phenex is to enable the effective division of labor within a collaborative curation workflow, specifically between tasks that do not require domain expertise and those that do. Research assistants used Phenex to enter the free-text descriptions for characters, character states, taxa, and voucher specimens; they also entered character-by-taxon matrices into Mesquite and exported these to NEXUS format for import into Phenex. Subsequently, ichthyological domain experts used Phenex to link each taxonomic name used in the publication to the current valid taxon name (as represented in the TTO), and finally composed EQ statements corresponding to each character–character state combination. The character-by-taxon matrix then automatically provided the mapping of EQ statements to individual taxa without further curator intervention being necessary.

**Figure 6:**
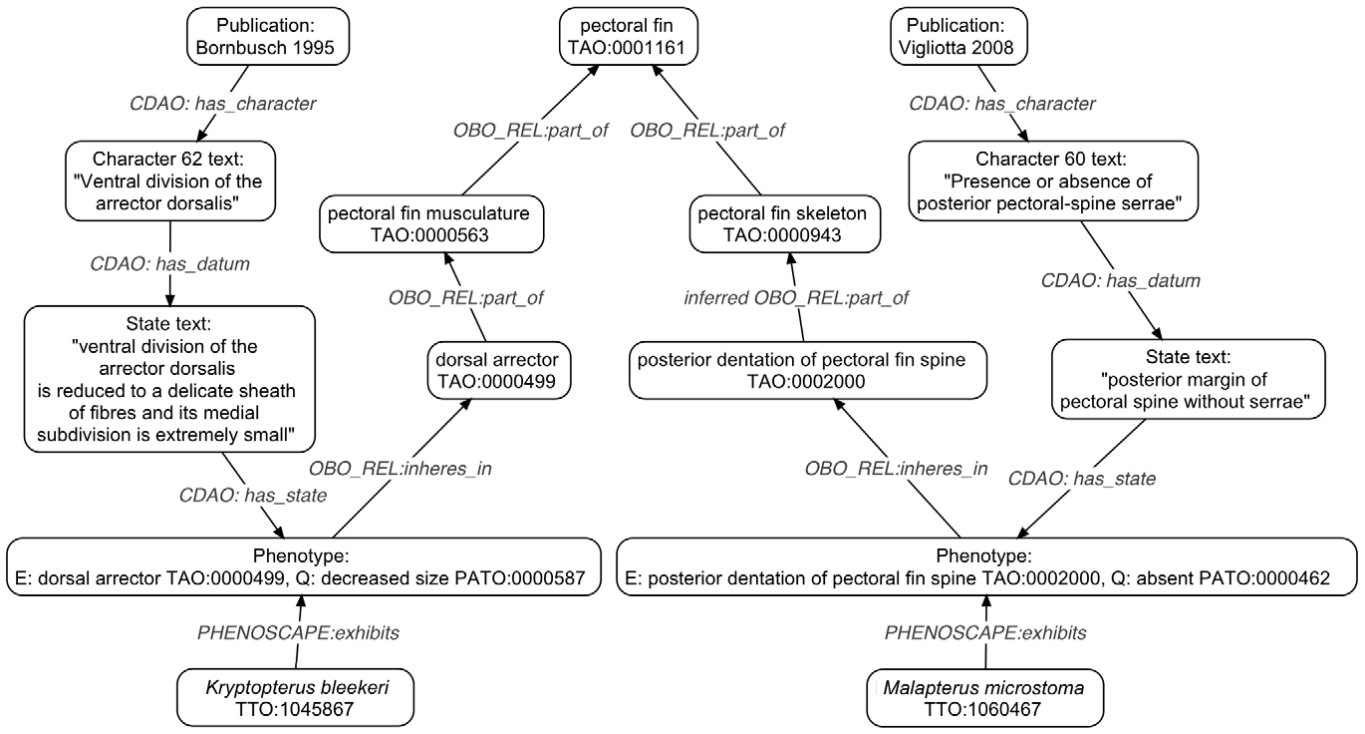
An example of lexigraphically dissimilar phenotype descriptions from two publications [32], [33] that are semantically similar in that they pertain to the same anatomical structure. The ‘dorsal arrector’ and the ‘posterior pectoral-spine serrae’ are both parts of the pectoral fin, which is immediately apparent to both humans and computers from the structure of the anatomy ontology. Some of the data relationships shown, such as *PHENOSCAPE:exhibits* and those from CDAO (Comparative Data Analysis Ontology, [30]), are not explicit in Phenex. Instead, these are generated by the interpretation of NeXML documents within the Phenoscape Knowledgebase data loading software.

The phenotype annotations generated in this way have been regularly exported from Phenex in its native NeXML data exchange format and subsequently imported into the Phenoscape Knowledgebase (KB) (http://kb.phenoscape.org). The Phenoscape KB is based on the Ontology-Based Database (OBD) [25], a software and data model specifically tailored for integrating and computing over ontology-annotated data. The logical structure of the ontologies, coupled with the deductive reasoning and the query interface provided by OBD, enable simple as well as powerful queries across phenotypes from both systematic studies of the Ostariophysi and genetic experiments in zebrafish. The Phenoscape KB demonstates that the EQ annotations created within Phenex can be amalgamated with data from multiple studies to reveal relationships that would be extremely difficult to discover without exploiting the structure of the ontologies (Figure 6).

## Discussion

A wealth of information about phenotypic diversity among taxa can be found in the scientific literature as text descriptions, but it can only realize a fraction of its value in that medium. The challenge facing evolutionary bioinformatics is how to efficiently put this information into a form that allows comparisons of data across studies, and allows linkages to relevant data from other sources, such as genetically characterized phenotypes, geographic localities, phylogenetic relationships, etc. User-friendly tools for curation of the systematic biology literature, such as Phenex, will be critical to the success of this effort. Annotating phenotypes and taxa from character-by-taxon matrices using ontologically defined terms and relations will open the door to a wide array of powerful ontology-driven applications for studying evolutionarily variable phenotypes.

While Phenex was designed in the context of a curation workflow for legacy data, the software may also be used to enter new character-by-taxon matrix data. While some initial training might be required to understand ontologies and the EQ model, constructing EQ statements at the time of entering characters and character states may be more efficient and accurate than *post hoc* curation. Because Phenex can be configured to load terms from any OBO ontology, it can be applied to data curation for any taxonomic group as long as appropriate anatomy and taxonomy ontologies exist. Employing Phenex for the creation of new datasets and within novel taxonomic groups will likely drive development of the Phenex user interface in ways that further increase the breadth of its applicability.

Even though ontologically annotated character-by-taxon matrices are tremendously useful for applications that compare data published by different authors at different times in different publications, using these data to construct a phylogenetic supermatrix [27] composed of EQ statements from multiple studies is inherently fraught with challenges. In particular, the dissociation of character states into EQs makes this difficult in two ways. First, the mapping between a character state and an EQ is not necessarily one-to-one, i.e., there may be multiple EQs for a single state, and this in and of itself precludes a simple substitution of EQs for character states in a matrix. Second, in the EQ formalism the *attribute* that forms part of a traditional character description is implicit in the hierarchical structure of the quality ontology (Figure 2), and hence inferring that two EQ descriptions represent alternative evolutionary states for a character is not straightforward. This can be advantageous, because EQ descriptions from unrelated studies are readily combined into a unified knowledgebase database, in contrast to the difficulty and uncertainty associated with combining characters from different character-by-taxon matrices. From this knowledgebase, similar taxon–phenotype annotations can be easily discovered by searching higher level anatomical or quality terms. On the other hand, the comparative context provided by a character-by-taxon matrix, in which phenotypes stand as alternative values for an evolutionary character, is not as readily apparent. It remains an open question how best to relate EQ phenotypes to alternative character states, and thus how to aggregate EQs into supermatrices for subsequent phylogenetic analysis.

### Future directions

A valuable future extension to Phenex would be to expand the ontology formats that it can utilize, such as the ability to load ontologies in the OWL standard (http://www.w3.org/TR/owl2-overview/), which is widely used in the computer science and semantic web community. However, the OBO fomat is still far more prevalent among biological ontologies, in large part due to its origins with the highly successful Gene Ontology [28], and so presently this is not a major limitation.

Aside from input formats, Phenex could be enhanced with the ability to directly export datasets in RDF-triple format (http://www.w3.org/RDF/), because in contrast to NeXML files standard RDF triples would be more readily integrated with other data resources on the Semantic Web [29]. Thanks to the development of the Comparative Data Analysis Ontology (CDAO) it is already straightforward to map NeXML data directly to CDAO concepts expressed in RDF [30]. Phenex could augment these data with ontology-based phenotype annotations.

Another advantage of Phenex exporting data directly as RDF triples is that it would make some of the semantics explicit that are currently only implied within the NeXML formatted export. For example, the *exhibits* links associating taxa and EQ phenotypes are not explicitly asserted in the NeXML export but instead must be constructed by custom programs that consume those files, such as the software that imports NeXML files into the Phenoscape KB. Similarly, the PhenoXML-encoding of a phenotype does not explicitly state the *is_a* and *inheres_in* relationships of a phenotype to the quality term and entity term, respectively. Making all semantics explicit will promote the greatest interoperability across diverse data sets.
